# Maize Stalk Obtained after Acid Treatment and Its Use for Simultaneous Removal of Cu^2+^, Pb^2+^, Ni^2+^, Cd^2+^, Cr^3+^ and Fe^3+^

**DOI:** 10.3390/polym14153141

**Published:** 2022-08-02

**Authors:** Nicoleta Mirela Marin

**Affiliations:** 1 National Research and Development Institute for Industrial Ecology ECOIND, Street Podu Dambovitei No. 57-73, District 6, 060652 Bucharest, Romania; nicoleta.marin@incdecoind.ro; 2 Science and Engineering of Oxide Materials and Nanomaterials, Faculty of Chemical Engineering and Biotechnologies University POLITEHNICA of Bucharest, Gh. Polizu 1-7, 011061 Bucharest, Romania

**Keywords:** copper, lead, nickel, iron, chromium, cadmium, natural polymer, low cost treatment

## Abstract

In this research, eco-friendly material represented by maize stalk (MS) obtained after acid treatment was employed for simultaneous removal of Cu^2+^, Pb^2+^, Ni^2+^, Cd^2+^, Cr^3+^ and Fe^3+^ (M^X+^) from simulated textile aqueous matrix and tannery wastewater produced by the leather industry. The acid treatment of MS was done with 4 M HCl. The influence of experimental parameters was evaluated in order to optimize the adsorption process for simulated textile matrix. The contact time 10–60 min and initial concentration of 0.5–1 mg/L M^X+^ influence were studied by batch method. Additionally, the adsorption data of M^X+^ onto MS was fitting by kinetic and isotherm models. The results obtained showed that the 60 min was necessary to reach adsorption equilibrium of the MS. The adsorption capacity of MS was 0.052 mg Cu^2+^/g of MS, 0.024 mg Pb^2+^/g of MS, 0.042 mg Ni^2+^/g of MS, 0.050 mg Cd^2+^/g of MS, 0.056 mg Fe^3+^/g of MS and 0.063 mg Cr^3+^/g of MS at pH = 4.2. The Langmuir model described the adsorption process very well. The MS showed huge selectivity for Cr^3+^ and Fe^3+^ in the presence of Cu^2+^, Pb^2+^, Ni^2+^ and Cd^2+^. The adsorption of M^X+^ from liquid phases were analyzed by spectrometric adsorption method (AAS). The solid phases of MS before and after adsorption by TG and SEM analysis were characterized. When MS was used for removal of M^X+^ from tannery wastewater, two major issues were investigated: First, the decrease of M^X+^ content from highly polluted and difficult to treat tannery wastewaters by improve its quality and in the second part, specific recovery of M^X+^ from MS mass increasing the economic efficiency of metals production based on green technology.

## 1. Introduction

Nowadays, the textile and leather industry produces significant volumes of wastewater with high concentrations of metals. Heavy metals are used in the production of pigments [1]. Over time, the demand for clothing and footwear has increased considerably. Thus, these industries contribute positively to the well-being of mankind but also have a negative effect on the environment if proper treatment operations are not applied [2]. Therefore, the monitoring of organic compounds and metal ions must be done before wastewater is discarded in an environmental aquatic medium [3]. For this, development of environmentally ecofriendly technologies could solve this problem. Biomaterials are a valuable alternative for the retention of metal ions and other organic compounds from effluents resulting from the painting and tannery process, respectively. Recently, several papers reported the capacity of bioadsorbents to remove heavy metals and other pollutants from polluted wastewater [4,5,6,7,8,9,10,11]. Biomaterials have been explored raw and after chemical modifications in order to enhance adsorption proprieties. Modifications of bio- materials can improve adsorption proprieties but can produce a secondary pollution if the dangerous chemical is applied for this aim. Taking into consideration this aspect is necessary to search for chemicals which induce a minimum impact to aquatic lives. Additionally, the most accessible pretreatment methods should be considered. The acid treatment enhances the adsorption characteristics of agricultural waste, mainly by the hydrolysis reactions. The influence of an activation process with HCl was investigated by BET analysis on untreated and treated Aloji clay. For this, the optimum conditions for height Pb^2+^ removal at 0.5 M HCl, T = 100 °C for 120 min was studied. By applying the above conditions, BET analysis highlighted the increase of surface area at 214.80 m^2^/g, pore volume of 0.1210 (cc/g) and pore size of 1.43 nm of HCl-activated Aloji clay compared with raw Aloji clay that has 138.7 m^2^/g, 0.0711 (cc/g) and 1.7 nm [12]. Vafakhah et al. studied Cu^2+^ removal from electroplating effluent solutions by corn cob and corn stalk raw and after being treated with 1 M HNO_3_ solutions. The corn stalk 40 g (70 mesh) was obtained after being oxidized with 200 mL HNO_3_ for 2 h. Corn stalk obtained after acid treatment has higher adsorption capacity reported at corn cob and corn stalk rested raw [13]. At the same time, alkaline treatment can be conducted. Bulgariu et al. studied the influence of alkaline treatment on the marine green algae for improving adsorption characteristics for removal of Zn^2+^, Co^2+^ and Pb^2+^. For this, a simple alkaline treatment was performed by mixing 5 g of algae bioadsorbent with solutions of 0.2–1 M NaOH for 24 h. The best removal of metal ions was obtained on algae treated with 0.6 M NaOH [14]. In this study, the adsorption behavior of the MS was studied in the presence of metal ions that is possible to be found in the effluents produced by the textile industry. Therefore, it is known that agricultural bioadsorbents are composed by lignin, cellulose and hemicellulose as major components and other functional groups, phenolic, carboxylic, alcohols, ketone and aldehyde [13]. These functional groups can ionize in aqueous solutions and act as a behavior of weak cation exchanger. Some possible theoretical explications for materials that can have low ion exchange behavior can be formulated as: If the solutions are concentrated in M^X+^, the adsorbent material has affinity for metal ions with low ionic radius and high charge. This behavior can be explained as follows: the higher ionic radius of M^X+^ decreases the speed of movement through the solution to the mass of the material. For metallic ions investigated in this paper: Fe^3+^ and Cr^3+^ have the lowest ionic radius, i.e., 0.55 and 0.62 Å followed by 0.69 Å Ni^2+^, 0.73 Å Cu^2+^, 0.95 Å Cd^2+^ and 1.19 Å Pb^2+^. The major goal of this research was to study the simultaneous removal of M^X+^ from simulated textile matrix onto modified maize stalk obtained after acid treatment. The kinetics of the adsorption process by pseudo-first-order and pseudo-second-order models was fitted. Additionally, the adsorption data obtained at equilibrium has been evaluated using Langmuir and Freundlich isotherm models. The surface morphology of MS by SEM images was evaluated. Thus, the MS adsorption behavior was investigated by a highly polluted tannery wastewater.

## 2. Materials and Methods

### 2.1. Chemicals

Standard solutions of 1000 mg/L Ni(NO_3_)_2_, Cd(NO_3_)_2_, Pb(NO_3_)_2_, Cu(NO_3_)_2_, Cr(NO_3_)_2_ and Fe(NO_3_)_3_ in HNO_3_ 0.5 mol/L, 37% HCl (density 1.16 g/mL) and 65% HNO_3_. Additionally, for calibration curves, the ICP multi-element standard containing XXI elements of 100 mg/L: As, Be, Cd, Ca, Cr, Co, Cu, Fe, Pb, Li, Mg, Mn, Mo, Ni, Sb, Se, Sr, Tl, Ti, V CertiPUR® was used and were purchased from Merck, Darmstadt, Germany. Acid Blue 113 (disodium; 8-anilino-5-[[4-[(3-sulfonatophenyl) diazenyl]naphthalen-1-yl]diazenyl]naphthalene-1-sulfonate) was purchased from Sigma Aldrich, Shanghai, China.

### 2.2. Equipment

In this study, atomic absorption spectrometer PinAAcle 900T (Perkin Elmer, Norwalk, CT, USA) has been used for determination of metal ion concentrations using air-acetylene flame mode. Concentrations of samples were detected after calibration with the spectrometer, with standards specific for each of the metal ions in the concentration range of 0.1–0.5 mg/L. 

Thermal analysis of solid MS mass before and after contact with simulated textile effluent were done on a STA 409 PC Luxx simultaneous thermogravimeter-differential scanning calorimeter TG/DSC (Netzsch, Selb, Wunsiedel, Germany). Additionally, solid phases of MS were quantified using scanning electron microscope (SEM) Quanta FEG 250 Fei, Eindhoven, The Netherlands. 

The XT220A Precise Gravimetrics scale, Dietikon, Switzerland was employed to weigh the MS masses. 

The pH of supernatant solutions before and after adsorption was monitored with the HI 255 pH meter, Hanna Instruments, Nijverheidslaan, Belgium. 

Ultrapure water of 18 MΩ/cm was obtained, with an Ultra-Clear system, Bremen, Germany.

### 2.3. Procedure for Obtained Shredded Maize Stalk Acid Treatment of MS

**Figure 1:**
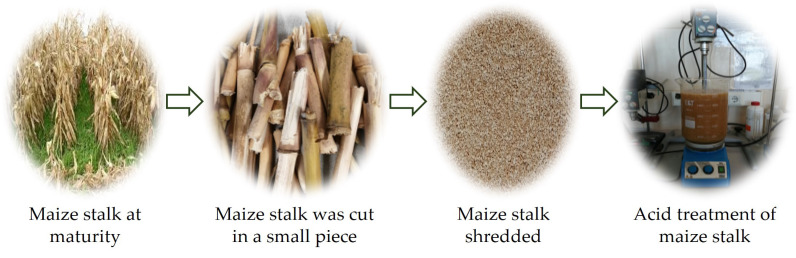
Schematic images for obtained shredded maize stalk applied for acid treatment.

MS was collected from Romanian Plain, after harvesting the corn. To obtain shredded maize stalk, the following steps were conducted. After collecting the stem, all the leaves were removed in the first step. Only the stem was processed to obtain biomass for adsorption studies. Subsequently, the stem was cut in small pieces and milled with an electric grinder. The procedure to obtaining shredded and acid treatment of MS are presented in Figure 1. The stalk was washed several times with tap water in order to remove dust, impurities and all accumulated microorganisms. Then, to obtain particle size to 1 mm, an electric grinder was used. The acid treatment of shredded MS was done by transferring the MS into 4 M HCl solutions in the proportion of 1–40 *w*/*v* and stirring at 100 rpm at room temperature for 8 h in a Berzelius glass. Subsequently, the maize stalk mass was washed with ultrapure water until the pH of supernatant became neutral (pH ≈ 6.5). The MS obtained was dried at room temperature (25 ± 2 °C) and kept in a desiccator throughout the experiments.

### 2.4. Metal Ions Used in Adsorption Studies 

For experimental studies, the retention of Cu^2+^, Pb^2+^, Ni^2+^, Cd^2+^, Cr^3+^ and Fe^3+^ were selected. Those M^X+^ induce a toxic effect to the aquatic environment and living organisms. Table 1 shows the main physical and chemical characteristics of the M^X+^ studied [15]. 

### 2.5. Validation Parameters of AAS Method 

From the stock solution of 100 mg/L of 21 metallic elements by dilution with 3% HNO_3_ solution, the standard solutions of 0.1, 0.2, 0.3, 0.4 and 0.5 mg/L were prepared. The absorbance corresponding to each metallic element were read at the wavelengths (λ) presented in Table 2 and the calibration curves were drawn based on the absorbances obtained against the concentration for each of the M^X+^ standard solutions. 

### 2.6. Preparation of Simulated Textile Matrices for Adsorption Studies

For adsorption studies, simulated textile matrices that contained metal ions in the range of 0.5–1 g/L and synthetic Acid Blue 113 dye in concentration of 100 mg/L in each sample were used. Metal ions existing in simulated textile samples were prepared from mono-element standard solutions by dilution with ultra-pure water to obtain concentrations of M^X+^. Only adsorption of M^X+^ was evaluated onto MS. 

### 2.7. Kinetic of Adsorption Experiments

Samples of 0.5 g MS were subjected to mechanical agitation for established optimum contact time into 250 mL Erlenmeyer flask. For this, the contact time was studied in the range of 10, 20, 30, 40, 50, 60, 70, 80 and 90 min, respectively. MS was shaken with 0.04 L of 0.5 mg/L solution that contained the studied M^X+^ and 100 mg/L Acid Blue 113, at 175 rpm (25 ± 2 °C) in Erlenmeyer flask. After every 10 min, the metal ion concentration from supernatant solutions by AAS was evaluated. All experiments were performed in duplicate and the value presented is the average of those.

The performance of the adsorption process was evaluated based on the adsorption capacity *Q_t_* (mg/g) which represents the mass of M^X+^ retained by one gram of MS at a time, *t* [17,18,19,20,21,22,23,24,25,26,27]:

(1) $$Q_{t}=\frac{\left(C_{i}-C_{t}\right)V}{m}$$

where *C_i_* and *C_t_* (mg/L) represent the concentrations of the supernatant before and after at time *t* of the adsorption process, *V* (L) the volume of samples to be tested, *m* (g) is the mass of the MS.

The Lagergren kinetic model was modulated by the following equation:

(2) $$log\left(Q_{e}-Q_{t}\right){=logQ}_{e}-\left(\frac{k_{1}}{2303}\right)t$$

and the second-order kinetic model, proposed by Ho and McKay, 1998 [18], was applied using the following equation:

(3) $$\frac{t}{Q_{t}}=\frac{1}{k_{2}Q_{e}2}+\frac{t}{Q_{e}}$$

where *k*_1_ is rate constant of Lagergren kinetic model and *k*_2_ is the pseudo-second-order rate constant of the M^x+^ adsorption onto MS mass.

### 2.8. Batch Adsorption Experiments

Samples of 0.5 g MS were stirred with 0.04 L simulated textile aqueous samples of metal ions that contained 0.5, 0.6, 0.7, 0.8, 0.9 and 1 mg/L Cu^2+^, Pb^2+^, Ni^2+^, Cd^2+^, Cr^3+^ and Fe^3+^ and 100 mg/L AB 113 in each metal ions concentration at 175 rpm (25 ± 2 °C) for 60 min in Erlenmeyer flask. The obtained mixtures were subjected to mechanical stirring for 60 min. After stirring, each sample was filtered and the concentrations of metal ions from supernatant were determined by AAS. All experiments were performed in duplicate and the value presented is the average of those.

The degree of pollutant removal R (%) was calculated with the following formula:

(4) $$R\;\left(\%\right)=\frac{C_{i}-C_{e}}{C_{i}}\times 100$$

where *C_i_* and *Ce* (mg/L) are the concentrations of the M^X+^ before and after adsorption process.

The quantity of the M^X+^ adsorption (*Q_e_*) by one gram of MS mass was determined by the Formula (5):

(5) $$Q_{e}=\frac{\left(C_{i}-C_{e}\right)V}{m}$$

### 2.9. Adsorption Experiments

Data obtained at equilibrium regarding adsorption of M^X+^ onto MS was studied by Langmuir and Freundlich isotherm models. The linear formula of the Langmuir and Freundlich models are: 

Langmuir

(6) $$\frac{C_{e}}{Q_{e}}=\frac{1}{bQ_{0}}+\frac{C_{e}}{Q_{0}}$$

(7) $$R_{L}=\frac{1}{1+bC_{0}}$$

Freundlich

(8) $$lnQ_{e}=lnK_{f}+\frac{1}{n}lnC_{e}$$

where *C_e_* (mg/L) is the equilibrium concentration of M^X+^, *Q_e_* are the adsorption capacity of MS at equilibrium and *Q_o_* (mg/g) is the maximum adsorption capacity of MS. *R*_L_ are the separation factor and indicates if the adsorption of M^X+^ onto MS is: favorable 0 < *R_L_* < 1, unfavorable *R_L_* > 1, linear *R_L_* = 1 or irreversible *R_L_* = 0. The *b* (L/mg) and *K_F_* (mg/g) are the Langmuir and Freundlich constants, and $\frac{1}{n}$ is an empirical parameter regarding intensity of adsorption.

### 2.10. Procedures for M^X+^ from Tannery Wastewater onto MS 

Wastewater resulting from the tanning of raw hides was depolluted using MS for the absorption of studied metal ions. For tannery wastewater, M^X+^ concentration and pH were determined before batch experiments. The experiments were carried out with 0.5 g MS (1 mm) that was added into Erlenmeyer flask and stirred with 0.04 L tannery wastewater at 175 rpm (25 ± 2 °C) for 60 min. At the end of the experiments, the mixture was filtered from which were determined the M^X+^ rezidual from liquid phases. All experiments were performed in duplicate and the value presented is the average of those. 

### 2.11. Procedures for Desorption Experiments

Desorption studies was conducted by adding 0.04 L of 4 M HCl over solid samples of MS obtained and described in Section 2.10. M^X+^ adsorption was from tannery wastewaters. The mixture was stirred 60 min at 175 rpm and then filtered. The liquid acid solutions were collected and Cu^2+^, Pb^2+^, Ni^2+^, Cd^2+^, Cr^3+^ and Fe^3+^ were determined by AAS. The M^X+^ desorption (*D* (%)) was evaluated using Formula (9).

(9) $$D\;\left(\%\right)\;=\frac{A}{B}\times 100$$

where *A* is the M^X+^ amount (mg) desorbed in liquid phases after desorption studies and *B* is the M^X+^ remaining (mg) onto MS mass after desorption studies. All experiments were performed in duplicate and the value presented is the average of those.

### 2.12. Characterization of Solid phases by TG and SEM 

TG curves were recorded using STA 409 PC Luxx thermogravimeter. Approximately equal quantity of samples was used for MS before and after adsorption and the analysis was performed in aluminum crucibles from ambient temperature 25 °C up to 1200 °C using a gradient speed of 10 °C/min.

## 3. Results and Discussion

### 3.1. Effect of Contact Time

**Figure 2:**
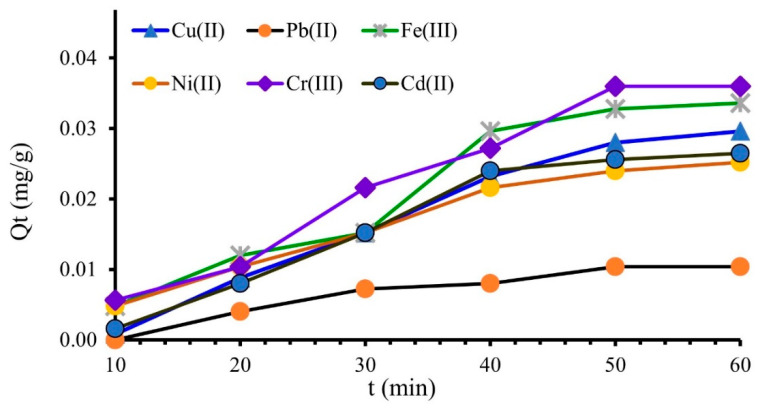
Influence of contact time on the retention of metal ions onto MS mass, 0.04 L of M^X+^ (*Ci* = 0.5 mg/L), contact time 10–60 min, 0.5 g MS, 175 rpm (25 ± 2 °C), pH = 4.2.

As can be seen in Figure 2, the M^X+^ adsorption increased in the first 40 min. From 40 up to 60 min, the adsorption process becomes slower and the adsorption values (*Qt*) increase insignificantly until equilibrium is reached. Based on these results and taking into account the porous structure of the MS, a contact time of 60 min was selected to evaluate the M^X+^ adsorption on the MS mass for the next adsorption experiments.

Similar results regarding influence of contact time by maize husk pretreated with tartric acid methanoic and phenol for Cu^2+^ removal were carried out. Applying batch adsorption experiments, the maximum removal of Cu^2+^ was obtained in 20–25 min for all materials pretreated [28]. Additionally, adsorption of Cr^3+^, Cd^2+^, Ni^2+^ and Cu^2+^ onto activated Teff Straw was investigated at different contact times. It was observed that the adsorption rate is faster at the beginning process and efficient removal was obtained in 60 min [1]. 

### 3.2. Effect of Initial Concentration 

**Figure 3:**
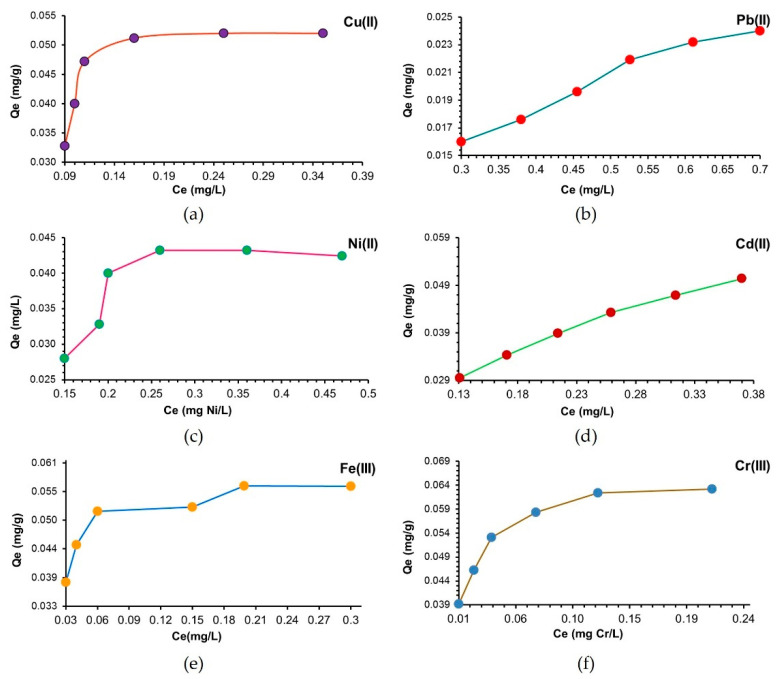
Effect of M^X+^ initial concentration on the adsorption capacity of MS. Experimental conditions: *Ci* = 0.5–1 mg/L M^X+^, contact time 60 min, 175 rpm (25 ± 2 °C), 0.5 g of MS, pH = 4.2.

The affinity of adsorption materials is defined as its ability to have a preference for certain ions in the presence of others found in the mixture solution. The adsorption isotherms of M^X+^ onto MS were determined using synthetic solutions of different concentrations that varied in the range of 0.5–1 g using a material dose of 0.5 g and a contact time of 60 min. Following these experiments, it was found that the adsorption capacity of MS increases with increasing concentration of M^X+^ (Figure 3). Thus, at higher concentrations than 0.8 mg Cu^2+^ and Ni^2+^, 0.9 mg/L Fe^3+^ and Cr^3+^ and 1 mg/L Pb^2+^ and Cd^2+^, the saturation level of the tested MS mass began to be observed. Thus, the adsorption capacity of MS was 0.052 mg Cu^2+^/g of MS, 0.024 mg Pb^2+^/g of MS, 0.042 mg Ni^2+^/g of MS, 0.050 mg Cd^2+^/g of MS, 0.056 mg Fe^3 +^/g of MS and 0.063 mg Cr^3+^/g of MS. As one can observe, good adsorption was obtained for Cr^3+^ and Fe^3+^.

**Figure 4:**
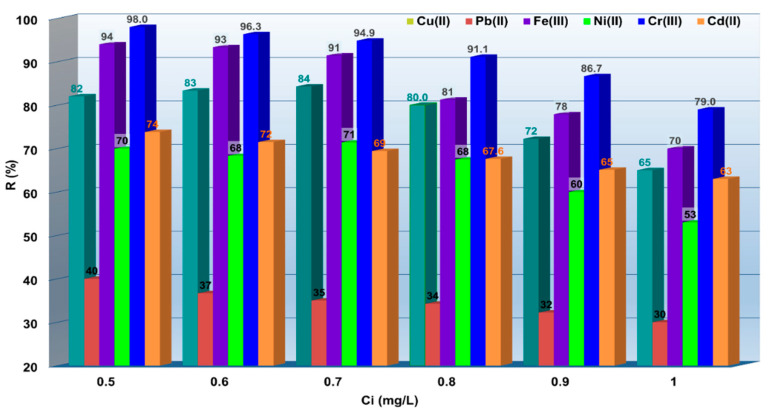
Removal of M^X+^ from simulated textile wastewater onto MS. Experimental conditions: *Ci*=0.5–1 mg/L M^X+^, contact time 60 min, 175 rpm, 0.5 g MS, pH = 4.2.

Moreover, the experimental data shows that with the increase of the metal ions concentration in the range studied, a decrease of the percent removal (*R*, %) was observed (Figure 4). As one can observe, *R* (%) decreased as: from 82% up to 65% for Cu^2+^, from 40% up to 30% for Pb^2+^, from 94% up to 70% for Fe^3+^, from 70% up to 53% for Ni^2+^, from 98% up to 79% for Cr^3+^ and from 74% up to 63% for Cu^2+^, respectively. This behavior can be explained as follows: the increase of M^X+^ quantity is dependent on the increase of M^X+^ initial concentration. Additionally, the percentages of M^X+^ retained on MS decrease as the number of functional groups are involved in the adsorption process. According to Ruchi et al., Cd(II) was removed by the modified Cucumis sativus peel (CSP) with HCl treatment. The removal efficiency at pH = 5 was obtained to be 85% for 20 mg/L Cd(II) [29].

The obtained data revealed that the MS material has efficient adsorption capacity for M^X+^ removal from simulated textile wastewater for concentrations in the range studied and in applying previously experimental conditions.

### 3.3. Kinetic Studies

**Figure 5:**
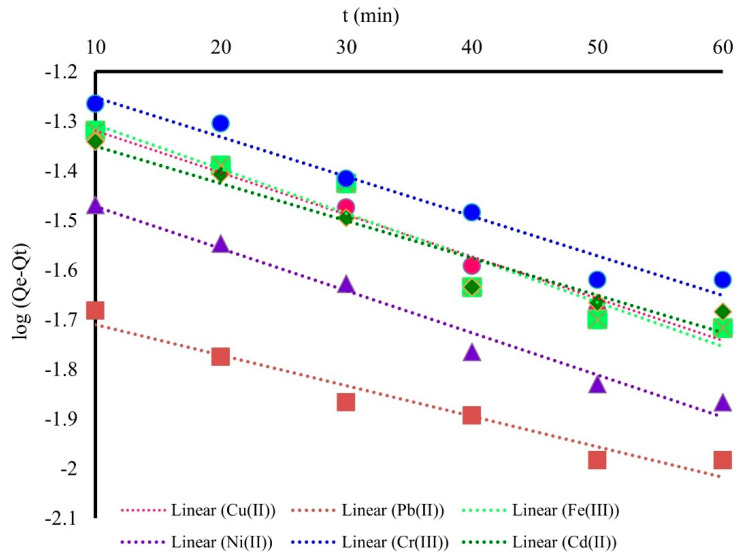
Graphical representation of pseudo-first-order kinetic model.

The experimental results regarding the correlation of the adsorption capacity (*Q_t_* (mg/g) according to the contact time (*t* (min)) were processed based on the pseudo-first-order and pseudo-second-order kinetics models [30]. Thus, if the adsorption process of M^X+^ onto MS is subjected to the pseudo-first-order kinetic model, the plot of *ln(Q_e_ − Q_t_*) against *t* (min) is linear. Based on the linear regression studies (Figure 5), the kinetic constants of the pseudo-first-order kinetic model were calculated and are presented in Table 3. The height value of R^2^ suggest that the adsorption process take places at the interface between liquid and solid phases.

In 1995, Ho describes the sorption of divalent ions on peat. The divalent metal ions were chemically bound by the functional groups of peat, such as aldehydes, ketones, acids and phenolic groups giving cation exchange properties of the peat. The adsorption process can be described by a second-order reaction, when the rate-limiting step is controlled by the ion exchange process between the functional groups of the tested material and the divalent metal ions. The sorption rate depends on the quantity of divalent ions adsorbed by the peat surface at time t and at equilibrium [18,31,32]. The pseudo-second-order constants (Table 4) were determined from linear regression line obtained representing the experimental data of *Q_t_*/*t* (min g/mg) against *t* (min). Recently, pseudo-second-order model has been used to describe the pollutants’ adsorption in corn silk/zeolite-Y adsorbent with R^2^ = 0.9991 and chemisorption controlling rate of the adsorption process [33]. Rice straw biochar was obtained after being modified with FeCl_3_·6H_2_O and FeSO_4_·7H_2_O for improving Cr^6+^ adsorption efficiency. Pseudo-second-order expressed the best fitting experimental data with a higher R^2^ = 0.996. The kinetic data expressed that the Cr^6+^ onto modified green adsorbent surface was governed by chemisorption process [34].

The height values of correlation coefficients (R^2^) obtained for pseudo-first-order in contrast with low R^2^ obtained for the pseudo-second-order kinetic model indicates that adsorption of M^X+^ onto MS is described very well by the pseudo-first-order kinetic model.

### 3.4. Adsorption Studies 

Adsorption of M^X+^ onto MS was evaluated with Langmuir and Freundlich isotherm models [33,35,36]. The Langmuir isotherm model starts from the hypothesis that the surface of the adsorbent material is homogeneous and the adsorption of pollutants was conducted in a single layer. The Freundlich model starts from the hypothesis that the surface of the adsorbent material is heterogeneous and the adsorption of pollutants was carried out in multilayers. The high values of R^2^ obtained for the Langmuir model (Table 5) compared to R^2^ values of the Freundlich model (Table 6) suggest that the adsorption of M^X+^ was achieved in a single monolayer. Moreover, it is observed that the maximum adsorption capacities values *Q_m_* (mg/g) are closer to the experimentally determined values of *Q_e_* (mg/g) shown in Section 3.2.

### 3.5. Applications of Maize Stalk in Tannery Wastewater Treatment

**Figure 6:**
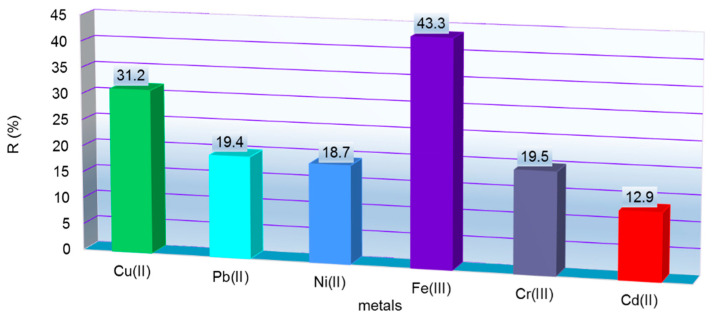
Removal of M^X+^ from tannery wastewaters onto MS mass. Experimental conditions: 0.04 L of tannery wastewaters that contained M^X+^ (*Ci* = 0.218 mg Cu^2+^/L, 0.618 mg Pb^2+^/L, 1.073 mg Ni^2+^/L, 0.163 mg Cd^2+^/L, 1006 mg Cr^3+^/L and 0.633 mg Fe^3+^/L), contact time 60 min, 175 rpm, 0.5 g MS, pH = 4.3.

Adsorption studies of metal ions existing in tannery wastewater onto MS by the batch method were performed. The concentrations of M^X+^ studied by AAS were determined to be: 0.218 mg Cu^2+^/L, 0.618 mg Pb^2+^/L, 1.073 mg Ni^2+^/L, 0.163 mg Cd^2+^/L, 1006 mg Cr^3+^/L and 0.633 mg Fe^3+^/L. The pH of the mixture (0.5 g MS with 0.04 L tannery wastewater) was also measured at the beginning (pH = 3.1) and after adsorption batch experiment (pH = 4.3). The obtained results of adsorption capacity (Q_e_) for M^X+^ from tannery wastewater onto MS were 15.68 mg Cr^3+^/g of MS, 0.022 mg Fe^3+^/g of MS, 0.016 mg Ni^2+^/g of MS, 0.010 mg Pb^2+^/g of MS, 0.005 mg Cu^2+^/g of MS and 0.002 mg Cd^2+^/g of MS. The selectivity of the MS can be described as follows, taking into consideration the Q_e_ values: Fe^3+^ > Cr^3+^ > Ni^2+^ > Pb^2+^ > Cu^2+^ > Cd^2+^. The degree of M^X+^ removal from tannery wastewater are presented in Figure 6.

An ideal bioadsorbent must have not only a height adsorption capacity but also the possibility to M^X+^ recovery from its mass. This operation involves the desorption of pollutants from bioadsorbent mass used in testing different striping agents [5]. The stripping agent is recommended so as not to damage the structure of the biomaterial and also due to a low cost of acquisitions.

**Figure 7:**
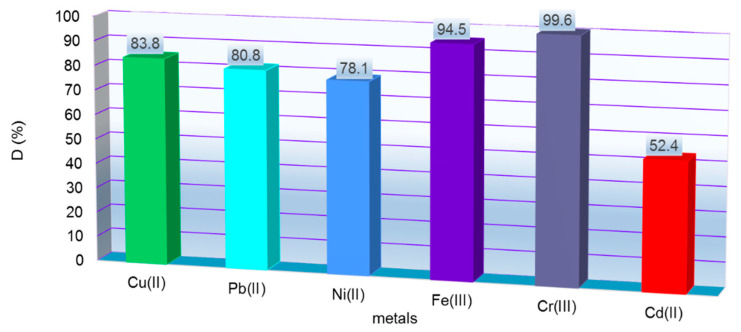
Desorption of M^X+^ from MS after adsorption process.

Applying the desorption conditions presented in Section 2.11, it is observed that the high concentration of desorption agent manages to desorb most of the M^X+^ retained onto MS mass: up to 99.6 % Cr^3+^ followed by 94.5 % Fe^3+^, 80.8 % Pb^2+^, 78,1 % Ni^2+^ and 52.4 % Cd^2+^ (see Figure 7). The desorption process was widely studied on various exhausted materials as follows. Basu et al. studied the influence of five stirring agents for lead recovery from exhausted biomass of cucumber peel. For this, 0.1 g of biomass loaded up to saturation with Pb^2+^ was mixed with 0.02 L of EDTA, alkaline salt (Na_2_CO_3_) and inorganic acid HCl, HNO_3_ and H_2_SO_4,_ for 3h (30 °C) at 120 rpm. Efficient desorption of Pb^2+^ from biomass was observed as HCl > HNO_3_ > EDTA > H_2_SO_4_ > Na_2_CO_3_ [27]. The desorption potential of 1M HCl was tested by Akpomie et al. for Mn^2+^ recovery and Ni^2 +^ from low-cost montmorillonite. Marin et al. studied regeneration of maize stalk exhausted with Cu^2+^ and Fe^3+^ with 3M HNO_3_. The metals were easy eluted from loaded maize and reused for five adsorption/desorption studies [37].

### 3.6. Characterization of MS before and after Adsorption

#### 3.6.1. Thermal Analysis

Numerous literature studies have presented the importance of thermal analysis to predict the mechanism of thermal degradation reported to mass losses for adsorbent materials and pollutants [38,39,40]. In this experiment, TG studies were performed in an oxidant atmosphere under dynamic conditions using a heating rate of 10 °C/min. Following the thermogravimetric analysis (TG), the significant mass losses can be observed for the basic macro-components of the MS. Thus, the first mass losses in the temperature range of 25–200 °C are attributed to the water existing in the porous structure as well as to the compounds without thermal stability. In the next step that occurred, a new degradation in the range 200–400 °C can be attributed to hemicellulose. Another mass loss was obtained between 400–600 °C and can be attributed to the degradation of cellulose. Lignin pyrolysis can be seen in the range of 600–800 °C [17].

**Figure 8:**
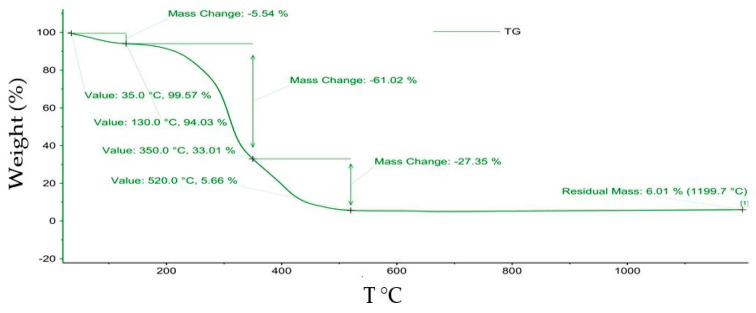
Curve of TG regarding maize stalk before adsorption process.

**Figure 9:**
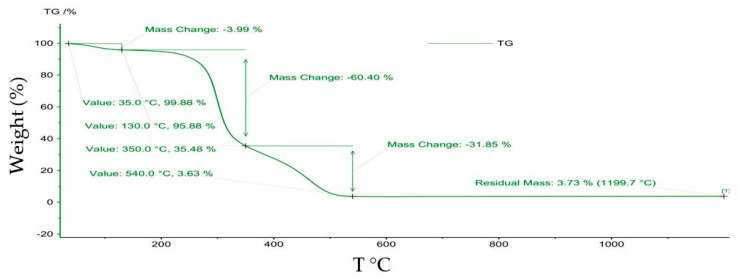
TG curve of maize stalk after adsorption process.

The residual masses of MS samples were 6.01% for MS blank and 3.73% for MS obtained after adsorption process, see Figure 8 and Figure 9. Additionally, the total mass loss for MS obtained after adsorption process was 96.27% which was larger than 94.2% obtained for MS before adsorption (Table 7). This suggested quantitative retention of M^X+^ onto MS mass.

#### 3.6.2. SEM Analysis

**Figure 10:**
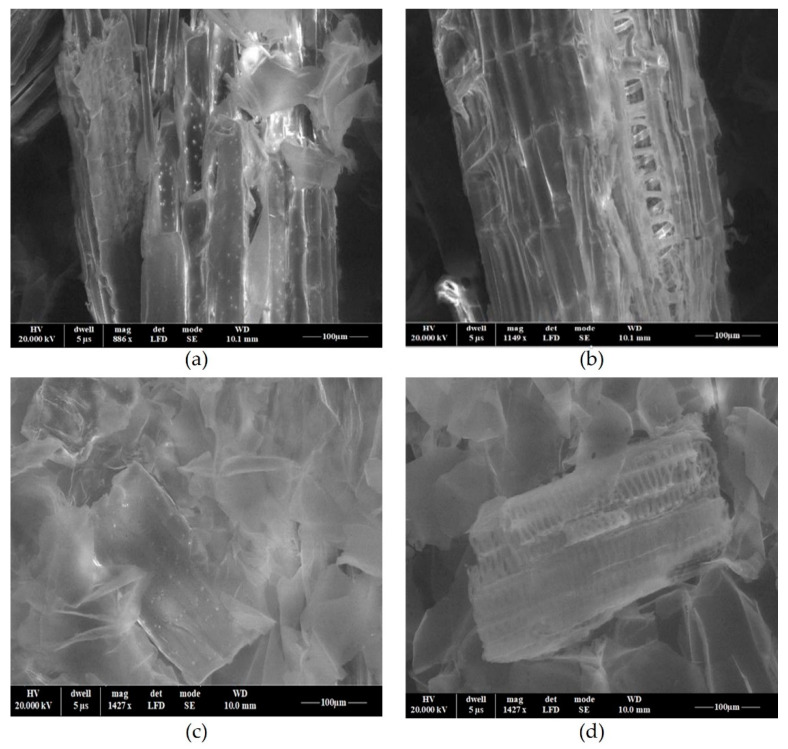
SEM images of MS at 1147 × (**a**), 886 × (**b**), 1427 × (**c**,**d**) magnification before (**a**,**b**) and after (**c**,**d**) adsorption.

The surface morphologies of maize stalk before and after M^X+^ adsorption were evaluated with a scanning electron microscope. The SEM images of MS at 1147 × (Figure 10a), 886 × (Figure 10b) revealed the porous structure of maize stalk before acid treatment. Thus, the porous structure favors the adsorption of the studied pollutants (Figure 10a,b). As can be seen in the MS image after adsorption, the surface of the material becomes smoother which suggests the retention of pollutants in the structure of the MS shown in Figure 10c,d.

## 4. Conclusions

In this paper, MS obtained after acid treatment was used as a natural polymer and an environmental eco-friendly material for removal of, i.e., Cu^2+^, Pb^2+^, Ni^2+^, Cd^2+^, Cr^3+^ and Fe^3+^ hazardous metal ions from simulated textile and tannery wastewater. For evaluating the adsorption of M^X+^ onto MS, 60 min was sufficient to reach the saturation of MS using the following conditions: 0.04 L of 0.5 mg/L each M^X+^ and 0.5 g MS at 175 rpm (25 ± 2 °C), pH = 4.2. Fitting the experimental data obtained at influence of contact time by pseudo-first-order and pseudo-second-order models shows that the M^X+^ adsorption is obeyed to pseudo-first-order model taking into consideration R^2^ values obtained. Based on the experimental data for MS adsorption when it is used as a solution that contained M^X+^ by the same concentration (1 mg/L M^X+^), the MS affinity was: Cr^3+^ > Fe^3+^ > Cu^2+^ > Cd^2+^ > Ni^2+^ > Pb^2+^. The Langmuir and Freundlich isotherm models of M^X+^ adsorption onto MS were evaluated. The R^2^ values were in accordance with the Langmuir model and predict that adsorption was achieved in a monolayer by the MS surface. In addition, TG analysis showed good stability of MS material if the adsorption process took place at various temperatures and also in the presence of pollutants in the mass of the MS obtained after the adsorption process. MS removed from tannery wastewater of all M^X+^ studied in different proportions in special Cr^3+^ represents the major pollutant of aqueous matrices studied. Even if the adsorptive capacity of maize stalk is not very high for Cu^2+^, Pb^2+^, Ni^2+^, Cd^2+^, Cr^3+^ and Fe^3+^, the low cost for obtained MS, together with its adsorptive ability, can offer a promising alternative for the treatment of wastewater. Therefore, the MS obtained from agricultural waste is safe for the environment and is a promising green material for removal of metal ions from polluted textile and tannery wastewater.

## Figures and Tables

**Table 1:** Characteristics of M^X+^ studied.

Metal	Copper	Lead	Nickel	Iron	Chromium	Cadmium
**Atomic number**	29	82	28	26	25	48
**Atomic mass (g/mol)**	63.5	207.2	58.7	55.8	51.9	121.4
**Oxidation states**	+2, +1	+2, +4	+2, +3	+3, +2	+6, +3	+2
**Atomic radius (Å)**	1.57	1.81	1.62	1.72	1.85	1.71
**Ionic radius (Å)**	0.73 (+2)	1.19 (+2)	0.69 (+2)	0.55 (+3)	0.62 (+3)	0.95 (+2)

**Table 2:** Linearity parameters of AAS method.

M^X+^	λ (nm)	Calibration Curves	R^2^	LOQ (µg/L)
Cu^2+^	232.75	y = 0.1376x + 0.0012	0.9999	3.5
Pb^2+^	283.31	y = 0.0203x − 0.0015	0.999	4
Ni^2+^	232	y = 0.0753x − 0.0042	0.9998	2.7
Fe^3+^	248.33	y = 0.0772x − 0.0016	0.9995	6.5
Cr^3+^	358.87	y = 0.0181x − 0.00009	0.9991	2.3
Cd^2+^	228.8	y = 0.4324x − 0.002	0.9998	3.1

LOQ (µg/L) represents limit of quantification of AAS method determined by applying methodology described in previous research [16].

**Table 3:** Pseudo-first-order model constants for adsorption of M^X+^ onto MS.

	Pseudo-First-Order Model					
Metal Ion	Cu^2+^	Pb^2+^	Ni^2+^	Cd^2+^	Cr^3+^	Fe^3+^
*Qe* (mg/g)	17.140	44.460	24.333	18.828	14.870	16.508
*k_1_* (min^−1^)	0.021	0.014	0.024	0.017	0.019	0.022
R^2^	0.9855	0.9454	0.9769	0.9430	0.9628	0.9333

**Table 4:** Pseudo-second-order constants for adsorption of the MX+ onto MS.

	Pseudo-Second-Order Kinetic Model					
Metal Ion	Cu^2+^	Pb^2+^	Ni^2+^	Cd^2+^	Cr^3+^	Fe^3+^
*Qe* (mg/g)	0.096	0.040	0.060	0.570	0.230	0.200
*k_2_* (g/mg min)	0.051	0.320	0.210	0.002	0.015	0.021
R^2^	0.2915	0.7411	0.9390	0.2000	0.2769	0.2484

**Table 5:** Langmuir constants for adsorption of the M^X+^ onto MS.

	Langmuir Isotherm Model					
Metal Ion	Cu^2+^	Pb^2+^	Ni^2+^	Cd^2+^	Cr^3+^	Fe^3+^
R^2^	0.9712	0.9828	0.9376	0.9992	0.9998	0.9985
*Q_m_* (mg/g)	1.258	0.085	0.526	0.346	8.058	4.405
*b*	0.048	0.484	0.102	0.240	0.008	0.013
*R_L_*	0.9970	0.9890	0.9960	0.9880	0.9992	0.9990

**Table 6:** Freundlich constants for adsorption of the M^X+^ onto MS mass.

	Freundlich Isotherm Model					
Metal ion	Cu^2+^	Pb^2+^	Ni^2+^	Cd^2+^	Cr^3+^	Fe^3+^
R^2^	0.6065	0.9800	0.6283	0.9962	0.944	0.7934
*Kf* (mg/g)	1.3	33.9	16.7	11.7	11.6	14.3
*1*/*n*	2.60	0.51	0.33	0.52	0.16	0.15
*n*	0.40	1.95	3.00	1.93	6.16	6.78

**Table 7:** Thermal weight loss for maize stalk before and after adsorption.

Thermal Behavior of Maize Stalk			
Before Adsorption		After Adsorption	
T °C	Weight (%)	T °C	Weight (%)
T_i_–35	–*	T_i_–35	0.03
35–130	5.5	35–130	4.0
130–350	61.2	130–350	60.4
350–520	27.4	350–540	31.9
T_i_–1200	94.2 **	T_i_–1200	96.3 **

Weight (%) = weight loss during the thermal degradation, –* no mass loss recorded, **(%) = total mass loss during the thermal degradation, T_i_ = initial temperature. TG curve was recorded as mass loss (%) against the T (°C).

## Data Availability

The data presented in this study are available upon request from the corresponding author.
